# Food and Drug Administration (FDA) Approvals of Biological Drugs in 2023

**DOI:** 10.3390/biomedicines12091992

**Published:** 2024-09-02

**Authors:** Alexander C. Martins, Mariana Y. Oshiro, Fernando Albericio, Beatriz G. de la Torre

**Affiliations:** 1 School of Health Sciences, UAM, Universidade Anhembi-Morumbi, São Paulo 03101-001, Brazil; mariyukie@hotmail.com; 2 Medical Information Department, Thermo Fisher Scientific, São Paulo 4542011, Brazil; 3 School of Chemistry and Physics, University of KwaZulu-Natal, Durban 4001, South Africa; 4 CIBER-BBN, Networking Centre on Bioengineering, Biomaterials and Nanomedicine, Department of Organic Chemistry, University of Barcelona, 08028 Barcelona, Spain; 5 KRISP, College of Health Sciences, University of KwaZulu-Natal, Durban 4001, South Africa; garciadelatorreb@ukzn.ac.za

**Keywords:** first approval, biologics, amyloid-related imaging abnormalities, Alzheimer′s disease, clinical trials, Food and Drug Administration, cancer, orphan drug, FDA approved, enzymes, monoclonal antibody, resistance

## Abstract

An increase in total drug (small molecules and biologics) approvals by the Food and Drug Administration (FDA) was seen in 2023 compared with the previous year. Cancer remained the disease most targeted by monoclonal antibodies (mAbs), followed by autoimmune conditions. Our data reveal the prevalence of approvals for biologics even during years when the total number of authorizations was low, such as in 2022. Over half the drugs that received the green light in 2023 benefited from expedited programs, as the incidence of many diseases increased. In addition, over half of the biologics approved received Orphan Drug Designation from the FDA. This narrative review delves into details of the most significant approvals in 2023, including mAbs, enzymes, and proteins, explaining their mechanisms of action, differences from previous drugs, placebo, and standards of care, and outcomes in clinical trials. Given the varying number of drugs authorized annually by the U.S. health authority, this review also examines the limits of external influences over the FDA′s decisions and independence regarding drug approvals and withdrawals.

## 1. Introduction 

The COVID-19 pandemic has now ended. This global health crisis brought about new perspectives, approaches, challenges, and outcomes. In 2022, as the world began to move past the pandemic, the U.S. Food and Drug Administration (FDA) continued to approve new drugs. However, the total number of drug approvals was low, with only 37 (including both small molecules and biological drugs) receiving the green light that year. This number is lower compared with previous years. In contrast, a different scenario emerged in 2023, with a total of 55 new drugs receiving authorization. The upward trend of antibody approvals continues, as in past years, with cancer still being the disease most targeted by monoclonal antibodies (mAbs), followed by autoimmune diseases [1,2,3]. 

In fact, in 2022 the approvals of biologics did not fall. In this regard, of the 37 new drugs approved, 15 were biologics, a similar figure to that registered for biologics in previous years.

The FDA seeks to promote a more agile approval process for drugs intended to treat serious diseases through four main expedited programs, namely, Priority Review, Breakthrough Therapy, Fast Track, and Accelerated Approval [4,5], thereby helping to deliver new therapies and support unmet medical needs faster. In 2023, as in previous years, several biologics were approved through these programs and also through Orphan Drug Designation. Regarding these expedited approaches for serious conditions, 36 out of the total 55 approvals in 2023 benefited from one or more of these programs [5].

Similar to previous years, the progress and promise in combating rare diseases continue to be defining characteristics of biological drugs. In 2023, there was a notable increase in the granting of Orphan Drug Designation status, marking it as one of the years with the highest number of designations compared with previous ones.

Another biologic to treat Alzheimer′s disease was approved, and diseases, such as Pompe disease, Fabry disease, Chaple disease, cancers with few treatment options, such as Nasopharyngeal Carcinoma, and an impressively high number of metastatic or recurrent (M/R) and relapsed or refractory (R/R) types of cancer are also targeted by the biologics authorized by the FDA in 2023.

Of all the biologics approved from 2015 to 2023, the most frequent targets include amyloid-β, Tumor Necrosis Factor (TNF), Programmed Cell Death Protein 1 (PD-1), CD-3, CD20, Human Epidermal Growth Factor Receptor 2 (HER2), Neonatal Fragment Crystalizable Receptor (FcRn), IL-23 p19 subunit and other interleukins, and calcitonin gene-related peptide, plus exogenous sources of enzymes [2,3,6]. Regarding the overall response rate (ORR), a valuable outcome measure, new biologics also demonstrated a higher ORR than the previous ones approved for the same therapeutic indication [7].

This narrative review provides a comprehensive and retrospective analysis spanning from 2015 to 2023. It is part of an ongoing series authored by the same individuals, delving into main themes and complexities within the pharmaceutical industry. It keeps the qualitative and quantitative analysis of the approved biologics as addressed in previous papers by the same authors. This review includes the analysis of external factors that may influence decision-making processes at the FDA and the perspectives of the biological drug market. It also provides a detailed look at the most important biologics approved in 2023 and their characteristics, such as the mechanism of action, and differences among other standards of care, efficacy, and safety. Biosimilars were excluded from the analysis.

## 2. Analysis of Biological Drug Approvals and External Influences over the FDA

The extent of external influence over decisions made by health authorities is unclear. Indeed, no major institution is free from such influences. Health authorities, for instance, base their decisions on factors beyond science as new evidence can emerge at any time. 

External influences include concerns over pollution, policy disagreements, political incentives, costs, and even religious values, some of which became evident during the COVID-19 pandemic [8]. These considerations allow for normative and political questions to impact the decisions made by health authorities [9]. In this context, the activities of the FDA are significantly affected by external and political influences. For example, the FDA′s budget is determined by Congress, and its decision-making capacity can be overridden by the Secretary of the Department of Health and Human Services (HHS) [8]. 

While the FDA is known for being science-oriented, it is also a political institution. This review focuses on the FDA′s Center for Biologics Evaluation and Research [10]. The influence of external factors was apparent in 2016, an election year in the U.S. with the lowest number of new drug approvals. When Trump took office in 2017, even with Trump having direct conflicts related to the FDA [11], we had a much higher total approval in that year; however, it is not clear how political and external influences have affected the biologics market, and further research into this matter is needed. In 2020 and 2021, during the height of the COVID-19 pandemic, the FDA authorized a high number of drugs. Although health authorities cannot make decisions solely based on scientific evidence, identifying the most influential factors is challenging. Despite these influences, 2023 was marked by a high number of approvals in the biologics market, with many orphan drugs receiving the green light. This indicates that the FDA′s decisions also consider unmet medical needs, disease severity, and the rising incidence of certain diseases [8]. Regarding the discussion on presidential influence, when a new president takes office, they possess the authority to alter the leadership of the Department of Health and Human Services and the Food and Drug Administration (FDA). However, it remains unclear how such changes impact the decisions of these health authorities or the ease with which these leadership changes can be implemented.

Table 1 illustrates the correlation between two key factors, provides a brief overview of their relationship, and sheds light on the quantitative analysis of annual drug approvals.

External influence in past years did not seem to affect the approval of biologics or Orphan Drug Designations (Table 2). Unmet medical needs have been the focus of pharmaceutical companies throughout the years as the prevalence of rare diseases, which are also emerging public health concerns, has increased [21,22]. Orphan drugs involve high investment and low returns for pharmaceutical companies, and they are therefore not financially attractive targets. Despite this consideration, of the biologics approved in recent years, Orphan Drug Designations have, on the whole, not fallen below 50% [23]. 

The Orphan Drug Act (ODA) of 1983 was intended to promote the investigation, research, and development of drugs for rare diseases, bringing about benefits for pharmaceutical companies, such as tax credits on research expenses, waived user fees, and prolonged orphan drug exclusivity for the respective drug [33]. The number of drugs granted this designation has increased over the past decades, with cancer being the most targeted area within all biologics receiving this status. When drugs other than biologics, such as small molecules, are considered, cancer is also the most targeted area for this FDA designation [34]. Importantly, regarding small molecules, there has also been a substantial increase in those addressing neurologic and pediatric-onset diseases over the past years [33,35]. It is clear the ODA has been effective in promoting the development of new drugs, thus adding to the arsenal of orphan drugs. The increased incidence of rare diseases has also influenced the FDA′s decisions.

## 3. Timeline for FDA-Approved Biologics

**Figure 1:**
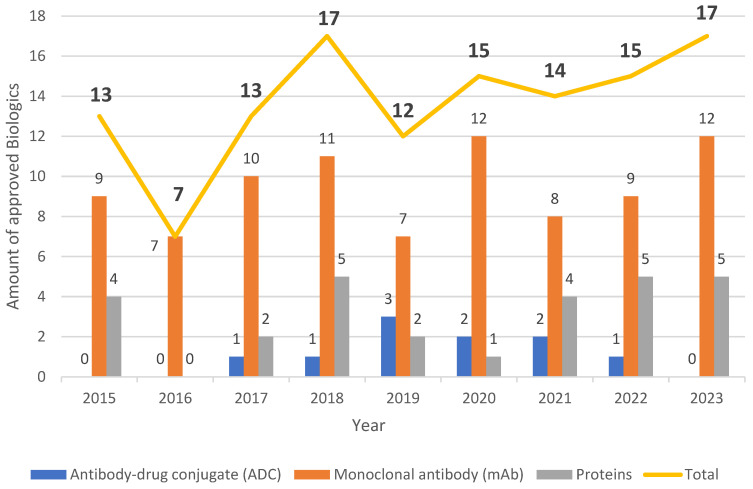
Biologics approved by the FDA from 2015 to 2023. This figure includes publicly available data from FDA databases such as The Purple Book, The Orange Book, and others.

Approximately nine mAbs have been authorized each year in the period covered by this review (2015–2023). The second year with the highest number of mAb approvals was 2023, after 2018, which registered the same number of biologics but one less mAb than in 2023 (Figure 1). Interestingly, in contrast with previous years, no antibody–drug conjugate (ADC) was approved in 2023 [36], and mAbs were the most approved biological class annually across the period.

**Figure 2:**
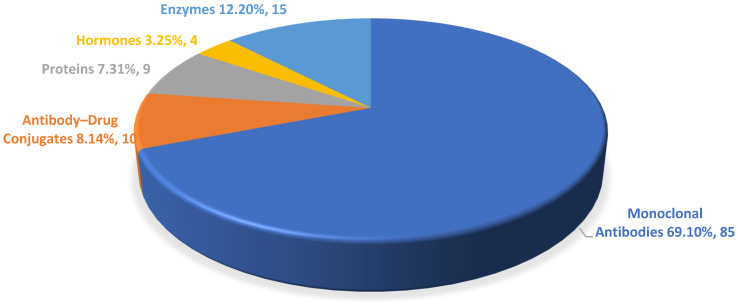
Percentage of new biopharmaceuticals approved by the FDA from 2015 to 2023. This figure includes publicly available data from FDA databases such as The Purple Book, The Orange Book, and others.

The mAbs authorized annually by the FDA accounted for over 50% of the total drug approvals over the period. In 2020, mAbs constituted 60% of the total biological approvals [3], and in 2023, mAb authorizations reached their highest percentage, with 12 out of all the 17 biologics being mAbs, accounting for 70% of the approvals. The percentage of proteins and enzymes to receive the green light increased in 2023, while ADCs decreased compared with previous years. However, several ADCs are in late-stage development phases [1,2] (Figure 2).

## 4. Alzheimer’s Disease—Lecanemab

In the context of neurodegenerative disorders, Alzheimer′s disease (AD) is the main form of dementia. It is characterized by the accumulation of proteins in the brain, such as intraneuronal neurofibrillary tangles (NFTs) of tau protein and extraneuronal amyloid-β (Aβ) plaques, cognitive decline, neuroinflammation, and disturbance of synaptic function [37,38]. Of note, there is some variation in the conformation of Aβ oligomers, which may predict their toxicity in a brain experiencing this kind of protein deposition. In this regard, new biologics for AD also have a distinct selectivity for certain forms of these proteins [39]. Between 2003 and 2021, there was no progress in drug development for this disease. However, in 2021, the FDA approved a breakthrough drug, namely, Aduhelm^TM^ (aducanumab), the first human mAb targeting the root cause of AD and considered the biologic of that year [2]. More than two years later, Leqembi^TM^ (lecanemab), also used for treating AD, was introduced to the market (Table 3). 

Lecanemab is the most recent breakthrough in the fight against AD. It is a humanized mAb, described as a biological drug directed against aggregated soluble and insoluble forms of Aβ with increased selectivity for protofibrils than for fibrils [42,43,44]. It was authorized under accelerated approval in the U.S. [40,45]—a route that expedites the approval of drugs intended to treat unmet medical needs [46]. Observation of a reduction in brain Aβ from baseline in patients treated with lecanemab vs. no reduction in placebo patients supports the accelerated approval granted [47]. 

Drugs previously authorized for the treatment of AD were unable to slow down the progression of the disease [48,49]. Given that the efficacy of many drugs used to treat this condition is compromised when crossing the blood–brain barrier because of pharmacokinetic issues, the choice of treatment for dementias and other diseases of the brain is strongly determined by side effects vs. benefits, among other factors [50]. The most common adverse events reported to date for the AD biologics lecanemab and aducanumab include amyloid-related imaging abnormalities (ARIA), ARIA-H (showing hemorrhage), microhemorrhage, and headaches, with lecanemab demonstrating a lower incidence of ARIA-E (showing edema and effusion), better safety, and greater reduction in Aβ levels than that achieved with other mAbs still undergoing clinical trials, such as gantenerumab, donanemab, and solanezumab [38,40,41,51,52,53]. ARIA is a common adverse event of treatments with mAbs.

Lecanemab has analogous characteristics to aducanumab, including classification as an IgG1 mAb and a target population comprising patients in the early stages of AD who have tested positive for β-amyloid pathology. The mechanism of action of these two drugs also involves the reduction in Aβ plaques. Both exhibit binding affinity to Aβ oligomers (ABO). However, lecanemab shows additional binding capacity to protofibrillar Aβ aggregates, whereas aducanumab targets fibrillar aggregates. These drugs also differ in parameters such as half-life and titration schedule [38,45,54,55]. Lecanemab and aducanumab also have some drawbacks, such as the need for patients to undergo Magnetic Resonance Imaging (MRI) scans before treatment, and also for follow-up purposes during treatment, varying the MRI monitoring schedules for each mAb. Also, these drugs can be administered only as intravenous infusions, lecanemab being given once every two weeks and aducanumab once every four weeks [41,51]. Of note, most biologics on the market for diseases other than AD are administered via subcutaneous injection, which is an advantage compared to intravenous infusions. Lecanemab and aducanumab reinforce not only the amyloid hypothesis in AD [56,57] but also provide hope for AD patients after decades devoid of breakthroughs in the field [2,58].

Clinical trials with lecanemab conducted on AD patients with mild cognitive impairment or mild dementia gave promising results [59,60]. The recommended dosage in the prescribing information is 10 mg/kg administered once every two weeks, aligning with the findings from clinical trials where it was identified as the effective dose of 90% (ED90). ED90 estimates the dose that achieves a 90% effective clinical outcome in the population under study [45]. However, results from the same study indicated that the primary endpoint of achieving a minimum reduction in clinical decline, measured by the Bayesian-designed Alzheimer′s Disease Composite Score (ADCOMS) at 12 months from baseline, was not met. The study can be found on clinical.trials.gov ID NCT01767311. However, in the same study, the secondary endpoint, which was a change from baseline at 18 months, indicated a reduction in brain amyloid at the recommended dosage of 10 mg/kg. In addition, MRI results pointed to hippocampal volume loss at the same dosage, which may be related to Aβ clearance [45,61]. A meta-analysis and other studies in the literature also support the statistically positive results found in clinical trials, which include but are not limited to ADCOMS, AD Assessment Scale–Cognitive Subscale (ADAS-Cog), Clinical Dementia Rating Sum of Boxes (CDR-SB), and amyloid PET Standardized Uptake Volume Ratio (SUVr). In patients with mild or early AD, the meta-analysis demonstrated that lecanemb statistically slows down the outcome measures, having positive effects on cognition [38,42]. Of note, autopsy findings revealed cerebral hemorrhages and histiocytic vasculitis with necrotizing vasculopathy, showing amyloid deposition within blood vessels in a 65-year-old patient who received three doses of lecanemab during a Phase 3 clinical trial. The patient had a stroke 4 days after the last administration of lecanemab. Other reports of death during clinical trials, as well as cases of brain swelling, among other severe events, raise questions regarding the safety of the drug [42,62,63]. Further research into the safety profile of mAbs indicated for the treatment of AD is necessary. 

## 5. Enzymes and Proteins

Genes that encode proteins responsible for lysosomal activities are important to maintain some body functions. The mutation of these genes may cause a deficiency in such proteins, which in turn may lead to the accumulation of glycosphingolipids, glycogen, cholesterol, and oligosaccharides, among others, that may trigger a variety of clinical manifestations. Lysosomal storage disorders (LSDs), for which there is no cure, result from a deficiency in lysosomal proteins. According to the National Organization for Rare Disorders (NORD) in the U.S., there are around 50 of these enzyme deficiencies. In this context, since the ODA in 1983, the FDA has approved over 500 orphan drugs, which include biological and non-biological drugs. Most of the therapeutic options for these deficiencies are intended to lower the accumulation of substances by increasing the cellular activity of these enzymes [64,65]. Some of these diseases and new treatment options approved in 2023 are shown below in Table 4.

## 6. Non-Central Nervous System Manifestations of α-Mannosidosis—Velmanase-α

Lamzede^TM^ (velmanase-α) was the first approved biological drug for a rare disease in 2023. It is indicated for both pediatric and adult patients with α-mannosidosis, a lysosomal storage disorder caused by a mutation in the MAN2B1 gene [66,72]. As seen in previous years, enzymes—almost always orphan drugs—are commonly authorized as replacement therapies [2,3]. Velmanase-α is the first enzyme replacement therapy (ERT) indicated for the lack of the α-D-mannosidase enzyme caused by α-mannosidosis. ERT is a common approach and has been effective in improving several symptoms of some diseases. However, it may lack effectiveness in others, including neurological conditions, for instance. Similar to AD, LSDs may worsen as the population ages. Given greater life expectancies, there is a need for advancements in treatments for LSDs [32,67].

## 7. Fabry Disease—Pegunigalsidase-α

Fabry disease is still considered a rare disease, and it is one of the most relevant LSDs. It can trigger the accumulation of substrates such as glycosphingolipids in the body, leading to chronic pain, cardiac issues, neuropathic pain, and renal damage, among others. It is caused by a reduction in α-galactosidase-A [68,73]. In 2023, the biologic Elfabrio^TM^ (pegunigalsidase-α) was authorized for the treatment of Fabry disease. This drug is a hydrolytic lysosomal neutral glycosphingolipid-specific and pegylated enzyme produced in tobacco cells that provides an exogenous source of α-galactosidase-A [74,75]. This biologic meets key requirements to be classified as an orphan drug and demonstrates significant pharmacokinetic benefits and a favorable safety profile. However, it does not offer a clinical advantage over other ERTs already available on the market. Health authorities around the world encouraged the sponsors to provide further clinical data to establish relevant advantages and obtain orphan drug status, but have so far denied the Orphan Drug Designation [74]. There are only two other ERTs available, Replagal^TM^ (original approval date 3 August 2001 in Europe), Fabrazyme^TM^ (original approval date 24 April 2003), and the chaperone Galafold^TM^ (migalastat). Considering the many difficulties encountered in the fight against rare conditions, the authorization of a new orphan drug is welcomed. However, further research into pegunigalsidase-α is still required. Of note, this review addresses FDA-approved biologicals and does not cover those that have received authorization from other health authorities. In this regard, we clarify that Fabrazyme^TM^ received approval from the FDA in April 2003, but Replagal^TM^ did not demonstrate sufficient clinical benefits to be approved, thus receiving a negative response from the FDA in January 2001. However, this drug is available in many other countries [76].

## 8. Pompe Disease—Cipaglucosidase-α

Pompe disease is a rare hereditary lysosomal disorder resulting from a deficiency in the enzyme GAA (which is important for breaking down glycogen into glucose in the lysosome). This deficiency leads to the buildup of glycogen in different tissues [77], causing heart problems and muscle weakness.

Two main clinical presentations are typically identified as follows: infantile-onset Pompe disease (IOPD) and late-onset Pompe disease (LOPD). These presentations vary in age of onset, affected organs, and disease severity [78]. Infantile-onset Pompe disease, which occurs shortly after birth, is the most severe form, showing symptoms that include cardiomyopathy, respiratory failure, and skeletal muscle weakness. Conversely, late-onset Pompe disease progresses at a slower pace and affects mainly skeletal muscle [79].

ERTs have been available for Pompe disease for some years. Although this therapy has shown benefits such as improving heart health and developmental milestones in infants and slowing down disease progression in adults, it is important to note that it is not a cure. The disease continues to progress in both children and adults despite undergoing enzyme replacement therapy (ERT). Hence, there is a strong emphasis on discovering more effective enzyme treatments and exploring alternative approaches such as gene therapy or reducing specific substances to address Pompe disease more effectively [80].

Amicus Therapeutics was in the process of developing a long-term ERT called Pombiliti™ (Cipaglucosidase α). On 28 September 2023, the FDA approved Pombiliti™ and Opfolda™ (miglustat), a unique two-component therapy. Pombiliti^TM^ in combination with Opfolda^TM^, an enzyme stabilizer, is designed as a targeted treatment for adult patients facing late-onset Pompe disease. The suggested dosage for Pombiliti^TM^ is 20 mg/kg of body weight, and it is administered as an intravenous infusion every other week. During clinical trials, infusion-associated reactions (IARs) with Pombiliti™ were reported in 32% of patients. Of these, 3% experienced severe reactions, including symptoms such as pharyngeal edema and anaphylaxis. Most reactions were mild to moderate, but some led to treatment discontinuation, notably because of urticaria and hypotension [25,70,81].

Another ERT for Pompe disease is provided by Nexviazyme™, which was approved by the FDA in 2021 [2]. While both Pombiliti^TM^ and Nexviazyme™ have side effects, the former has more adverse reactions.

## 9. Pituitary Hormone Deficiency—Somatrogon

Pituitary hormone deficiency refers to an insufficiency of the pituitary gland, with Growth Hormone Deficiency (GHD) being the most prevalent form, occurring either congenitally or acquired [82,83,84]. In recent years, there have been only two new biological treatments for GHD, namely, Sogroya™ (somapacitan, approved in 2020 and initially authorized for adult patients, but later also approved for pediatric use) and Skytrofa™ (lonapegsomatropin, approved in 2021). Ngenla^TM^ (somatrogon) was approved in 2023 and is indicated for pediatric patients. Like lonapegsomatropin, somapacitan is indicated for pediatrics and adolescents. They were all produced using recombinant DNA technology, but through different methods: Somapacitan and Lonapegsomatropin were synthesized using *E. coli*, while somatrogon was produced in Chinese Hamster Ovary (CHO) cells [69,84,85,86].

These three biologics share many characteristics (Table 5), including common adverse reactions. However, somapacitan, which is indicated for both adult and pediatric patients, can have a more extended range of adverse reactions in adults, including arthralgia, sleep disorder, dyspepsia, dizziness, tonsillitis, hypertension, and back pain, among others. 

In terms of pharmacokinetics, these three drugs show similarities. Somatrogon has an estimated peripheral volume of distribution of 0.671 L/kg, somapacitan has volumes of 14.6 L/kg in adults and 1.7 L/kg in pediatrics, and lonapegsomatropin has 0.13 L/kg (estimated from the literature). Their half-lives are also very similar [69,85,86]. In a Phase 3 clinical trial compared with the first growth hormone treatment approved by the FDA in 1995 (somatropin), somatrogon demonstrated non-inferiority in the primary endpoint (height velocity) and was well tolerated in children [87]. This information can be found at ClinicalTrials.gov, study no. NCT02968004.

Ryzneuta™ (efbemalenograstim-α), first approved in China in May 2023 and, in the same year, by the FDA, is a prescription medication classified as a leukocyte growth factor. It is used to reduce the occurrence of infection, particularly febrile neutropenia caused by myelosuppressive anticancer drugs, in adult patients with non-myeloid malignancies [88,89].

Efbemalenograstim works by binding to specific receptors on hematopoietic cells. This interaction stimulates various processes within the cells, including proliferation, differentiation, commitment, and end-cell functional activation. The recommended dosage for Ryzneuta™ is a single subcutaneous injection of 20 mg administered once per chemotherapy cycle [71]. In placebo-controlled and Neulasta-controlled Phase 3 clinical trials, the mean duration of severe neutropenia was evaluated, demonstrating non-inferiority and lower duration of severe neutropenia compared with control interventions [90,91].

Table 6 provides a brief overview of the only biological drug approved for lower respiratory tract disease.

## 10. Cancer 

### 10.1. Rising Cancer Cases

According to the World Health Organization and the International Agency for Research on Cancer, in 2022, there were about 20 million cancer cases and 9.7 million deaths globally. After diagnosis of cancer, around 53.5 million people presented an average life expectancy of five years. By 2050, it is expected that there will be over 35 million cases of cancer, which represents a 77% increase from the estimated 20 million cases in 2022. This increase is mainly due to increased life expectancy, and factors such as air pollution, lifestyle (smoking and alcohol consumption), and obesity are driving the increased incidence of cancer [95].

The American Cancer Society, which estimates the number of new cases of cancer and deaths caused by cancer per year in the U.S., foresees 2,001,140 new cancer cases and 611,720 cancer deaths in 2024. However, mortality from this disease has continued to decline since 2021, and over 4 million deaths have been prevented since 1991 because of reductions in smoking, earlier detection of certain types of cancer, and improved treatment options for both early-stage and metastatic cancers.

The incidence rates for six of the top ten cancers increased annually from 2015 to 2019. Breast, pancreas, and uterine corpus cancers increased by 0.6% to 1%, and prostate, liver (female), kidney, human papillomavirus-associated oral cancers, and melanoma by 2% to 3%. Cervical cancer (ages 30–44 years) and colorectal cancer (ages < 55 years) also presented annual increases of 1% to 2% among young adults [96].

### 10.2. Biologics Approved for Cancer Treatment in 2023

Cancer was the most targeted disease by the drugs approved by the FDA in 2023. In this regard, 6 of the 17 biologics approved were indicated for cancer treatment. All of these drugs are mAbs, and four of them are bispecific. Of note, all six biologics are indicated for metastatic or recurrent and/or relapsed or refractory cancer. The latest bispecific mAbs approved by the FDA are indicated for cancer. The highest number of approvals of bispecific mAbs to date occurred in 2023 (Table 7).

## 11. Merkel Cell Carcinoma (MCC)—Retifanlimab

MCC is a rare but serious form of aggressive skin cancer that can metastasize. It is a cutaneous neuroendocrine tumor that tends to appear more often in older individuals, typically around 50 years of age [110,111]. In recent decades, researchers have become increasingly interested in understanding how MCC develops, especially its connection to the Merkel cell polyomavirus. It seems that most cases result from the virus, which causes cells to become cancerous. However, a minority of cases are believed to be caused by DNA damage from exposure to ultraviolet radiation [111].

The treatment for a tumor typically involves surgical removal, often followed by radiation therapy. If there is lymph node involvement, a combination of lymph node dissection and radiation therapy may be necessary. Metastatic MCC is typically treated with immune checkpoint inhibitors like avelumab and pembrolizumab [3,112]. In this regard, retifanlimab (Zynyz^TM^) received the green light from the FDA on 22 March 2023 for the treatment of M/R locally advanced MCC in adults [12,31].

Retifanlimab is a humanized IgG4 kappa mAb and belongs to a class of drugs that bind to either the programmed death receptor (PD-1) or the PD-ligand (PD-L1), thus blocking the PD-1/PD-L1 interaction. When PD-L1 and PD-L2 bind to the PD-1 receptor on T-cells, they slow down T-cell multiplication and cytokines release. This increase in PD-1 ligands is observed in certain tumors, and it hampers the ability of T-cells to actively monitor and attack cancer cells. Retifanlimab steps in by attaching to the PD-1 receptor, disrupting its interaction with PD-L1 and PD-L2 ligands, thereby boosting T-cell activity [99,113].

Regarding other treatments for MCC, on 23 March 2017, Bavencio^TM^ (avelumab) was the first product to receive FDA authorization for this type of cancer [2,114].

Retifanlimab and avelumab target different proteins. While retifanlimab inhibits the PD-1 protein, avelumab attaches to PD-L1, preventing it from interacting with its receptors PD-1 and B7.1, and by doing so, this interruption allows the immune response to overcome the inhibitory effects of PD-L1, leading to the restoration of its ability to fight tumors [99,113,114,115].

Avelumab is used as a first-line therapy in some countries, but retifanlimab is considered a better option for monotherapy in advanced MCC. Both biologics are associated with good outcomes and are well tolerated. However, no studies have yet directly compared the two drugs. Adaptive mechanisms of MCC can lead to resistance to immunotherapies, suggesting a potential trend in future research in this area.

## 12. Diffuse Large B-Cell Lymphoma (DLBCL)—Epcoritamab

DLBCL, which can be challenging to diagnose because of the requirement for multiple tests to classify it accurately, accounts for approximately 24–30% of all non-Hodgkin′s Lymphomas (NHLs) [116,117,118]. Moreover, a high rate of patient response to standard treatment has been reported; over half of the patients can achieve remission with systemic therapies like rituximab, cyclophosphamide, doxorubicin, vincristine, and prednisone (R-CHOP). However, for cases such as high-grade B-cell lymphoma involving genetic rearrangements or refractory cases, these therapies may prove insufficient, necessitating more advanced therapies [116,119,120,121]. Of note, rituximab is a chimeric mAb that was first approved in 1997, and in 2017, it was authorized with a modification with a human hyaluronidase injection, targeting CD-20 [2].

Two new approvals of bispecific mAbs indicated for DLBCL occurred in 2023, namely, Epkinly^TM^ (epcoritamab) and Columvi^TM^ (glofitamab). Regarding the challenges in treating DLBCL, epcoritamab emerges as a new line of treatment for high-grade B-cell lymphoma after two or more systemic therapies. Importantly, other bispecific mAbs for DLBCL treatment are currently in development [99,100,116,121,122,123,124,125]. Glofitamab and epcoritamab are similar to each other in many aspects, sharing the most common adverse reactions and mechanisms of action. However, they differ in cycle of treatment, exposure parameters, and the Volume of Distribution (VD): glofitamab has a VD of 5.6 L and a half-life of 7.6 days, while epcoritamab has a VD of 25.6 L and a half-life of 22 days (58%) [100,101]. A comparison of epcoritamab with a non-bispecific biologic approved in 2020 for the same therapeutic indication, namely, tafasitamab [3], revealed a better VD (25.6 L) and a half-life (22 days) for epcoritamab vs. tafasitamab (VD 9.3 L and half-life of 17 days) [100,126]. Thus, the differences between non-bispecific mAbs and bispecific ones are remarkable, with the latter demonstrating advantageous pharmacokinetics.

## 13. Multiple Myeloma (MM)—Elranatamab

MM encompasses a spectrum of plasma cell malignancies characterized by various translocations, mutations, and cytogenetic changes. Despite MM being a distinct diagnosis, its classification involves different subtypes. It is one of the most prevalent hematologic diseases characterized by abnormal cell proliferation in the bone marrow. Key biomarkers such as elevated BCMA and GPRC5D levels are commonly observed in MM patients [109,127,128,129,130,131]. 

The numerous therapies currently available to treat this disease (thalidomide, lenalidomide, pomalidomide, bortezomib, carfilzomib, mAbs such as elotuzumab and daratumumab, corticosteroids, and transplants, among others) have led to an improvement in progression-free survival (PFS). However, it is unclear where the peak of these improvements begins or if it has already been reached. Therefore, different approaches are required to better understand myeloma biology, and/or additional trials are required to assess the need for changes in current treatments [2,127,128,132,133].

Although survival rates have improved in recent decades, almost all MM patients relapse, which makes the condition more difficult to treat despite advancements in therapies for R/R patients since the 1990s [128,131]. In 2023, new approvals for R/R MM [12,103,106,127] support the recent trend in drug authorizations for the treatment of this disease.

Elranatamab was approved by the FDA in 2023 through Orphan Drug Designation, Fast Track, and Breakthrough Therapy programs [1]. It is a humanized bispecific mAb whose mechanism of action involves binding to BCMA on plasma cells, MM cells, and plasmablasts and also binding to CD3 on T-cells, inducing T-cell-mediated on MM cells [105,106,107]. In a Phase 1 clinical trial, elranatamab demonstrated a 70% overall response rate (ORR) with a 30% complete response (CR) in the efficacious dose range and an ORR of 83% at the Recommended Phase 2 Dose [128,134]. In a Phase 2 trial, elranatamab was well tolerated by patients. Patients in the regimen of 76 mg once a week (QW) did not present immune effector cell-associated neurotoxicity syndrome (ICANS) or cytokine release syndrome (CRS). However, a significant number of patients reported infections, mostly of the respiratory tract [128,134,135,136].

In 2023, the FDA also approved talquetamab, a highly selective humanized bispecific mAb that binds to the GPRC5D receptor and CD3 receptors expressed on the surface of MM cells, inducing T-cell activation and degranulation of CD4+ and CD8+ T-cells, leading to the death of MM cells [137]. This biologic was developed by a giant in the pharma industry (Janssen Biotech) that has been granted authorization for many biologics for the treatment of MM in recent years, including its own bispecific mAb which, like the newly approved elranatamab [2,3] targets CD3 and BCMA. Both bispecific biologics are highly selective for their targets and show some differences, for instance in their pharmacokinetic profiles [104,105].

The discovery of new biomarkers, which are essential to combat cancer, may bring with them novel targets for biological drugs, like the approvals we have seen in recent years.

## 14. Nasopharyngeal Carcinoma (NPC)—Toripalimab

NPC is a very distinct type of cancer that is highly related to infection by Epstein–Barr virus (EBV). It differs in histology and epidemiology from other cancers such as head and neck cancers, and it is diagnosed often when it is already advanced [138,139,140,141]. Despite advances in modern treatments for this type of cancer, many patients experience recurrence and metastasis. Recent biologicals approved for head and neck cancer, such as pembrolizumab and nivolumab, do not include indications for NPC [2,139]. Results of clinical trials demonstrated that toripalimab (approved by the FDA in 2023) showed substantial positive outcomes in patients in terms of tolerability, safety, efficacy, ORR, and PFS [141,142].

## 15. Autoimmune Conditions

Table 8 presents all the drugs approved for autoimmune conditions in 2023. Let’s take a detailed look at each one.

## 16. Generalized Myasthenia Gravis (gMG)—Rozanolixizumab

MG is an autoimmune disease that affects proteins in the neuromuscular junction (AChR, MuSK, and Low-Density Lipoprotein Receptor Related Protein-4—LRP4) [148]. In this regard, the mAb rozanolixizumab (approved in 2023) is the second biological neonatal Fc receptor antagonist approved by the FDA for gMG, after efgartigimod (an engineered human IgG1 fragment), which was authorized in 2021 [2,149]. Efgartigimod, a fragment of mAb indicated for adults with gMG who are AChR antibody-positive, was one of the breakthroughs of that year [149]. Parallel to that biologic is the newly approved rozanolixizumab (a humanized IgG4 anti-FcRn mAb), demonstrating that drug development for gMG continues. Rozanolixizumab, unlike the drug approved in 2021, is the first biologic authorized for the treatment of gMG patients who are both AChR and MuSK antibody-positive [150,151]. Of note, there is no cure for gMG to date, and the development of FcRn receptor antagonists has been well received mainly because FcRn promotes gMG symptoms by transporting and protecting IgG from degradation and extending the half-life of MuSK and AChR autoantibodies. In this context, treatments to antagonize FcRn receptors emerge as an effective approach to tackle this condition [150,152,153,154].

Rozanolixizumab has shown effectiveness in a Phase 3 clinical trial in the Myasthenia Gravis-Activities of Daily Living (MG-ADL) score (*p* < 0.001) compared with a placebo. In a Phase 1 study [155], it demonstrated a 68% reduction in plasma IgG levels when administered either subcutaneously or intravenously. Phase 2 studies indicated that rozanolixizumab is well tolerated and effective, even for a different condition, namely, thrombocytopenia [151,156,157,158]. Notably, several companies are studying mAbs that also target the FcRn receptor [151]. Similarly, efgartigimod has shown a decrease in total IgG levels after the first administration in clinical trials [149,159,160]. Although Phase 3 studies reported issues such as discomfort, depression, and anxiety, this drug showed good results on measures such as the EuroQol 5 Dimension 5 Level (EQ-5D-5L) and Health-Related Quality of Life (HRQoL) [161]. For gMG, the common adverse reactions differ between the two biologics. In efgartigimod-treated patients, ≥ 10% experienced headaches and urinary tract infections. In rozanolixizumab-treated patients, ≥10% experienced headaches, infections, diarrhea, pyrexia, hypersensitivity reactions, and nausea [143,162]. 

Monoclonal antibodies are extensively researched because of their potential to treat diseases other than their primary approved target. As an example, rituximab, a human/murine chimeric mAb targeting CD20 proteins, was the first mAb ever approved for cancer. Initially approved by the U.S. FDA in 1997 for the treatment of non-Hodgkin′s lymphoma, rituximab continues to be used today [2,148]. Rituximab is the oldest example of a cancer drug being extended to autoimmune conditions. It was first approved to treat cancer and later authorized for autoimmune conditions, including rheumatoid arthritis [163]. An increase in off-label prescriptions of rituximab for autoimmune conditions has been noted over recent decades. These conditions include Multiple Sclerosis (MS) and MG, for which the data regarding MuSK-positive patients has been positive so far. However, AChR-positive patients’ data seem to vary considerably, and there is no clear evidence to support the off-label use of this drug in these patients [148]. Of note, ongoing clinical research addressing the use of rituximab for autoimmune conditions includes studies examining a wide variety of diseases and distinct administration routes. In this regard, a Phase 1 trial for intrathecal injections in MS patients is underway, which will be completed in December 2024 [164], as is a Phase 2 trial for Autoimmune Premature Ovarian Insufficiency [165] and a Phase 2 trial for Inflammatory Demyelinating Polyneuropathy, which will be completed in August 2027 [166], among others. No results have been reported to date for these studies.

## 17. Plaque Psoriasis—Bimekizumab

Psoriasis is an autoimmune, inflammatory, and chronic disease, that can evolve to the formation of plaques in the body. It can be painful and affect the patient not only physically, but also psychosocially [167]. Bimekizumab (Bimzelx^TM^) was approved by the FDA in 2023 based on evidence from two clinical trials conducted in many countries, plus two additional active-controlled clinical trials (superiority studies, equivalence studies, and non-inferiority studies compared standard-of-care treatments) for supportive data on safety. This drug is an anti-interleukin (IL)-17A and -17F, and it is used to treat adults with moderate to severe plaque psoriasis who are suitable candidates for systemic therapy or phototherapy [168].

Regarding other drugs approved by the FDA for this condition in recent years, such as secukinumab (2015), ixekizumab (2016), and brodalumab (2017) [2], bimekizumab stands out as the most recent IL-17 inhibitor available, a humanized monoclonal IgG1 antibody, because of its unique mechanism of action. It works by neutralizing both IL-17A and IL-17F, which is a different mechanism of action to that exerted byixekizumab and secukinumab, for instance, which selectively inhibit IL17A, and brodalumab, which acts as an IL17 receptor antagonist [2,169].

The two placebo-controlled trials (Ps-1 and Ps-2 Phase 2 trials), which are the basis for FDA approval, and the other two additional trials (one active-controlled trial (Ps-3) and one open-label extension trial) demonstrate the efficacy and safety of this new biologic. In Ps-1 and Ps-2 trials, when compared with a placebo, the majority of patients who achieved an Investigator Global Assessment (IGA score) of 0 (zero), meaning *Clear*, or 1 (one), meaning *Almost Clear*, was Ps-1 = 84% of patients achieved an IGA 0 or 1, and Ps-2 = 93% of patients achieved an IGA 0 or 1. The additional trials evaluating safety did not identify any new adverse reactions other than the most common ones (≥1%) described in the Prescribing Information for Bimzelx^TM^ [144,168,169].

## 18. Ulcerative Colitis (UC)—Mirikizumab

UC is a chronic inflammatory disease that affects the rectum and colon. The development of the condition is influenced by several factors, including genetics, problems with the epithelial barrier, abnormal immune responses, and environmental factors. Several treatments are available, including 5-aminosalicylic acid drugs and corticosteroids [170].

The development of new therapies is crucial because up to half of patients do not respond well initially, or eventually stop responding [171]. In October 2023, mirikizumab (Omvoh^TM^) received the green light from the FDA [12]. This drug is a humanized IgG4 monoclonal antibody that targets the p19 subunit of IL-23 cytokine. This binding prevents IL-23 from interacting with its receptor [145]. 

The approval came from results generated by the LUCENT program, which included two randomized, double-blind, placebo-controlled Phase 3 clinical trials. The program consisted of a 12-week induction study (UC-1) and a 40-week maintenance study (UC-2) involving patients who had previously tried other treatments, including biologics, without success or tolerance [170,171,172].

After 12 weeks of treatment with Omvoh^TM^, nearly 65% of patients had a clinical response and nearly 24% achieved remission, compared with 43% (clinical response) and 15% (clinical remission) for the placebo. For patients who achieved a clinical response at 12 weeks, Omvoh^TM^ proved to be effective across various patient groups, with 51% of all patients and 45% of those who had previously failed treatment with a biologic or Janus kinase inhibitor (JAKi) achieving clinical remission at one year, compared with the placebo group. Patients taking Omvoh^TM^ were less likely to stop treatment because of side effects, with rates of 1.6% in UC-1 and 1.5% in UC-2, compared with 7.2% in UC-1 and 8.3% in UC-2 for those on the placebo [145,172].

## 19. Conclusions

With the rise in chronic diseases worldwide, greater life expectancy, and increasing lack of response to current therapies, there is an increasing demand for more advanced and effective therapies. In this context, while the need for more individualized and highly selective therapies coupled with the urgent need for new biologics drive market growth, many other factors may influence the decisions made by the FDA.

The FDA, an agency under the Health and Human Services (HHS), has primary responsibilities, including regulating clinical investigations of drugs and ensuring the safety and efficacy of both human and veterinary drugs (small molecules and biological drugs). However, it may also be influenced by policies and regulatory issues beyond scientific evidence [8,173,174,175]. The FDA is recognized as a science-oriented agency that aims to protect the safety, rights, and welfare of participants in clinical trials. Despite this, the FDA and HHS differ in certain aspects of Human Subject Protection Regulations. For example, the FDA does not have special provisions for certain populations like pregnant women, children, or the elderly, while the HHS does. In addition, the FDA requires compliance from both the investigator and the sponsor of a study, whereas the HHS requires assurances and certifications from institutions. In some areas, such as criteria for Institution Review Board (IRB) approval and IRB membership requirements, the FDA and HHS are virtually identical. However, they differ in criteria for disqualifying an IRB or institution, considering different risk factors [175]. In terms of division of labor, scientists are expected to perform their tasks in a science-based manner, free from external burdens, while other officials tasked with duties other than scientific ones, set policies and guidelines, among other tasks. Nevertheless, every decision made by any individual associated with the FDA must be based on scientific evidence, must be transparent, and must serve the best interests of the population.

The possible influences that the FDA may encounter, as discussed in this paper, start from the period before a New Drug Application (NDA) [176] is submitted and continue until the drug is approved. However, when it comes to the process of discontinuing or withdrawing drugs that are already on the market, the decision appears to be more science-based. The FDA is the agency responsible for requesting post-market safety studies. Although health agencies often discontinue medications for various reasons, primarily related to safety concerns, it is uncommon for mAbs to be discontinued. [1].

The FDA facilitates the delivery of new therapies for unmet medical needs through a faster approval process for drugs intended to treat serious diseases. This is achieved via four distinct programs including Priority Review, Breakthrough Therapy, Fast Track, and Accelerated Approval [4]. In 2023, for instance, elranatamab was authorized for the treatment of MM, receiving Orphan Drug Designation under the Breakthrough Therapy program, which speeds up the review and development of drugs that have the potential to demonstrate better outcomes over existing therapies, and the Fast Track program, which is also designed to speed up and clear the way for drugs intended to treat serious conditions [1,4]. As the incidence of cancer increases, it becomes even more crucial to find ways to further reduce the time spent in the accelerated approval process for these types of drugs. In the period addressed (from 2015 to 2023). Cancer continued to be the most common disease targeted by drugs, followed by autoimmune conditions, and the most common targets included TNF, PD-1, CD3, CD20, HER2, FcRn, IL-23 p19 subunit, and other interleukins, and calcitonin gene-related peptide, plus an exogenous source of enzymes [2,3,6]. Regarding autoimmune conditions, the innovativeness among the approvals in 2023 is reflected by pozelimab, the first treatment for Chaple disease, an ultra-rare condition affecting less than 100 people worldwide [146,147]. The biologics approved in 2023 have achieved the expected outcomes in clinical trials, proving to be superior and/or non-inferior to previous therapies or placebos. No ADC was approved in 2023, but several ADCs are in late-stage development; therefore, next year new ones may be added to the therapeutic arsenal against cancer (tusamitamab ravtansine and datopotamab deruxtecan) [1].

Increasing efforts to combat rare diseases have become a noticeable trend. In 2023, over half of the approved biological drugs received the Orphan Drug Designation. When looking at all drugs approved in 2023, including both small molecules and biologicals, more than half (28 out of 55 approved drugs) also received the Orphan Drug Designation [5].

The rise in Orphan Drug Designations granted in recent years suggests that the FDA may continue to increase approvals for both small molecules and biologics. This trend also highlights the emerging medical need for treatments for rare diseases. The FDA has been successful in encouraging pharmaceutical companies to tackle specific therapeutic areas, which is evident in the innovativeness of some new drugs, the rise in engineered mAbs approved in 2023, and the increase in drug authorizations this year. Notably, 2023 was one of the years with the highest total number of approvals, the highest number of bispecific mAbs, and a significant number of approvals benefiting from the expedited programs.

To highlight the importance of biosimilars, thousands of rare diseases and other conditions still do not have approved treatments. As more biological drugs are authorized, more biosimilars will become available in the future. These biosimilars could be much cheaper for patients and governments alike. In addition, the availability of biosimilars could drive down the prices of the original reference drugs, thereby benefiting even more patients [34,177].

As discussed in this review, biological drugs are characterized by their high target selectivity and associated significant side effects. However, because of their high selectivity, they tend to produce fewer and generally milder adverse reactions compared with chemotherapies. Chemotherapies, being non-specific, debilitate the patient’s immune system, increasing the risk of infections. In contrast, other cancer treatments such as radiation therapy, although localized, can cause, for instance, severe skin reactions and may even lead to the development of secondary cancers. The evaluation of adverse reactions in new biologic drugs is crucial, not only for meeting regulatory requirements and facilitating the approval process but also for balancing risks against therapeutic benefits, thereby optimizing the overall efficacy of this drug class. A deeper understanding of this aspect of drugs promotes the customization of treatments, enhancing the effectiveness and safety of new personalized and more selective therapies, such as biologics.

In conclusion, despite the conflicts of interest and potential threats to the FDA′s independence, it is crucial to emphasize the health-oriented mission of the agency. The FDA must serve the best interests of the country, addressing the growing medical needs of the population. Therefore, solid guidelines, policies, and other documents guiding the FDA′s decisions must be grounded primarily in science. The scientific basis is essential to ensure adaptation to the evolving nature of diseases, such as conditions becoming increasingly refractory to a wide variety of treatments, or the relapse of cancer patients. These challenges call for the development of new and advanced treatments.

## Figures and Tables

**Table 1:** Correlation of total drug approvals vs. biologics approvals by the FDA.

Year	Total Drugs Approved (Biologics and NCEs)	Biologics Approved	References
2023	55	17 (30.9%)	[12]
2022	37	15 (40.5%)	[13]
2021	50	14 (28%)	[14]
2020	53	15 (28.3%)	[15]
2019	48	12 (25%)	[16]
2018	59	17 (28.8%)	[17]
2017	46	13 (28.2%)	[18]
2016	22	7 (31.8%)	[19]
2015	45	13 (28.8%)	[20]
2015–2023	415	123 (29.6%)	

This table includes publicly available data from FDA databases such as The Purple Book, The Orange Book, and others. Abbreviation: NCE—New Chemical Entity.

**Table 2:** Orphan Drug Designations granted by the FDA from 2015 to 2023.

Year	Biologics Approved	Orphan Drug Designations Granted for New Biologics
2015	13	7 (53%)
2016	7	2 (28%)
2017	13	5 (38%)
2018	17	13 (76%)
2019	12	9 (75%)
2020	15	10 (66%)
2021	14	7 (50%)
2022	15	7 (53%)
2023	17	9 (52.9%)
2015–2023	123	60 (48.78%)

This table includes publicly available data from FDA databases such as The Purple Book, The Orange Book, and the Search Orphan Drug Designations and Approvals database of the U.S. Department of Health and Human Services [3,24,25,26,27,28,29,30,31,32].

**Table 3:** New biologics for Alzheimer′s disease approved by the FDA in 2023.

Drug Name	Class and Administration	Mechanism of Action	Original Approval Date	Pharmaceutical Company	Therapeutic Indication
Leqembi^TM^ (lecanemab-irmb) [12,40,41]	Humanized mAb (IgG1) (intravenously)	Acts against aggregated soluble and insoluble forms of amyloid-β peptide	6 January 2023	Eisai, Inc., Nutley, NJ, USA, and Biogen, Cambridge, MA USA	Alzheimer′s disease

This table includes publicly available data from FDA databases such as The Purple Book, The Orange Book, and others. Abbreviations: IgG—Immunoglobulin Gamma; and mAb—monoclonal antibody.

**Table 4:** Enzymes and proteins approved by the FDA in 2023.

Drug Name	Class and Administration	Mechanism of Action	Original Approval Date	Pharmaceutical Company	Therapeutic Indication
Lamzede^TM^ (velmanase α -tycv) * [12,32,66,67]	Recombinant human enzyme (intravenously)	Catalyzes the degradation of accumulated mannose-containing oligosaccharides	16 February 2023	Chiesi Farmaceutici S.p.A, Parma, Italy	Non-central nervous system manifestations of α -mannosidosis
Elfabrio^TM^ (pegunigalsidase α -iwxj) [12,68]	Hydrolytic lysosomal neutral glycosphingolipid-specific enzyme (intravenously)	Provides an exogenous source of α-galactosidase	9 May 2023	Chiesi Farmaceutici S.p.A., Parma Italy	Fabry disease
Ngenla^TM^ (somatrogon-ghla) * [12,29,69]	Human growth hormone analog (rhGH) (fusion protein) (subcutaneously)	Binds to the GH receptor, leading to an increase in serum concentration of IGF-1	27 June 2023	Pfizer Ireland Pharmaceuticals, Ringaskiddy, Ireland	Pediatric patients who have growth failure
Pombiliti^TM^ (cipaglucosidase α -atga) * [12,25,70]	Recombinant human acid α -glucosidase (exogenous source of GAA) (intravenously)	Binds to the CI-MPR in skeletal muscle	28 September 2023	Amicus Therapeutics, Philadelphia, PA USA	Late-onset Pompe disease
Ryzneuta^TM^ (efbemalenograstim α -vuxw) [12,71]	G-CSF (recombinant fusion protein) (subcutaneously)	Stimulates the differentiation of stem cells, increasing neutrophils	16 November 2023	Evive Biotechnology Singapore PTE. LTD., Singapore	Neutropenia in adult patients receiving anti-cancer drugs

This table includes publicly available data from FDA databases such as The Purple Book, The Orange Book, and others. Abbreviations: (*)—Orphan Drug Designation granted by the FDA; rhGH—Recombinant Human Growth Hormone; GH—Growth Hormone; IGF-1—Insulin-Like Growth Factor 1; GAA—Acid Alpha-Glucosidase; and CI-MPR—Cation-Independent Mannose-6-Phosphate Receptor; G-CSF—Granulocyte-Colony Stimulating Factor.

**Table 5:** Differences and similarities in biological treatments for growth deficiency.

Biological Drug.	Dosage Forms	Most Common Adverse Reactions (≥5%)	Administration
Somatrogon	Prefilled pen	Pyrexia, injection site reactions, nasopharyngitis, headache, anemia, cough, vomiting, hypothyroidism, abdominal pain, rash, and oropharyngeal pain	Subcutaneous injection (once weekly)
Somapacitan		Pyrexia, nasopharyngitis, headache, pain in extremities, and injection site reaction	
Lonapegsomatropin	Prefilled cartridges (lyophilized powder, dual-chamber, prefilled cartridges, and diluent)	Pyrexia, viral infection, cough, nausea and vomiting, hemorrhage, diarrhea, abdominal pain, and arthralgia and arthritis	

**Table 6:** mAb for lower respiratory tract disease approved by the FDA in 2023.

Drug Name	Class and Administration	Mechanism of Action	Original Approval Date	Pharmaceutical Company	Therapeutic Indication
Beyfortus^TM^ (nirsevimab-alip) [92,93,94]	Recombinant human mAb (IgG1k) (intramuscular use)	Neutralizes RSV by inhibiting conformation changes in the F protein necessary for the fusion of the viral and cellular membranes and viral entry	17 July 2023	AstraZeneca AB, England, UK, and Sanofi, Paris, France	Prevention of RSV

This table includes publicly available data from FDA databases such as The Purple Book, The Orange Book, and others. Abbreviations: IgG1k—Immunoglobulin G 1 kappa; and RSV—Respiratory Syncytial Virus.

**Table 7:** Monoclonal antibodies for cancer approved by the FDA in 2023.

Drug Name	Class and Administration	Mechanism of Action	Original Approval Date	Pharmaceutical Company	Therapeutic Indication
Zynyz^TM^ (retifanlimab-dlwr) * [12,31,97,98]	Humanized mAb (IgG4) (intravenously)	Binds to the PD-1 receptor found on T-cells, blocking interaction with PD-L1 and PD-L2	22 March 2023	Incyte Corporation, Wilmington, DE USA	M/R locally advanced Merkel cell carcinoma
Epkinly^TM^ (epcoritamab-bysp) [12,99,100]	Humanized bispecific mAb (IgG1) (subcutaneously)	Simultaneously binds to CD3 on T-cells and CD20 on B-cells	19 May 2023	Co-developed by Genmab USA, Inc., Plainsboro, NJ, USA and AbbVie, North Chicago, IL, USA	R/R DLBCL (NOS), including DLBCL arising from indolent lymphoma, and high-grade B-cell lymphoma
Columvi^TM^ (glofitamab-gxbm) [12,101,102]	Humanized bispecific mAb (IgG1) (intravenously)	Simultaneously binds to CD3 on T-cells and CD20 on B-cells	15 June 2023	Genentech, Inc., South San Francisco, CA, USA	R/R DLBCL (NOS) or LBCL arising from follicular lymphoma
Talvey^TM^ (talquetamab-tgvs) * [28,103,104]	Humanized bispecific mAb (IgG4-PAA) (subcutaneously)	Acts as anti-GPRC5D heavy and light chain and anti-CD3 heavy and light chain	9 August 2023	Janssen Biotech, Inc., Beerse, Belgium	R/R multiple myeloma patients who have received at least four prior line therapies
Elrexfio^TM^ (elranatamab-bcmm) * [27,105,106,107]	Humanized bispecific mAb (IgG2Δa) (subcutaneously)	Binds BCMA on plasma cells, plasmablasts, and multiple myeloma cells and CD3 on T-cells	14 August 2023	Pfizer Inc., New York, NY, USA	R/R multiple myeloma patients who have received at least four prior lines of therapy
Loqtorzi^TM^ (toripalimab-tpzi) * [24,108,109]	Humanized mAb (IgG4K) (intravenously)	Binds to the PD-1 receptor and blocks its interaction with PD-L1 and PD-L2	27 October 2023	Coherus BioSciences, Inc., Redwood City, CA, USA	Adults with M/R NPC and as a single agent for the treatment of adults with M/Ru NPC

This table includes publicly available data from FDA databases such as The Purple Book, The Orange Book, and others. Abbreviations: (*)—Orphan Drug Designation granted by the FDA; IgG—Immunoglobulin; PAA—Proline–Alanine–Alanine; mAb—monoclonal antibody; PD-1—programmed death 1; PD-Ll/L2—programmed death ligands; M/R—metastatic or recurrent; DLBCL—Diffuse Large B-cell Lymphoma; R/R -relapsed or refractory; NOS—Not Otherwise Specified; CD—Cluster of Differentiation; LBCL—Large B-cell Lymphoma; anti-GPRC5D—G Protein-Coupled Receptor Class C Group 5 member D; IgG2Δ—Immunoglobulin 2-Alanine Kappa; M/Ru—metastatic or recurrent unresectable; and NPC—Nasopharyngeal Carcinoma.

**Table 8:** mAbs for autoimmune conditions approved by the FDA in 2023.

Drug Name	Class and Administration	Mechanism of Action	Original Approval Date	Pharmaceutical Company	Therapeutic Indication
Rystiggo^TM^ (rozanolixizumab-noli) * [12,30,143]	Humanized IgG4P mAb (subcutaneously)	Blocks FcRn, reducing the amount of free IgG	26 June 2023	UCB, Inc., Brussels, Belgium (Union Chimique Belge)	gMG in adult patients who are AChR or anti-MuSK antibody-positive
Bimzelx^TM^ (bimekizumab-bkzx) [12,144]	Humanized mAb (IgG1) (subcutaneously)	Binds to human IL-17A, IL17F, and IL-17-AF cytokines	17 October 2023	UCB, Inc., Brussels, Belgium (Union Chimique Belge)	Moderate to severe plaque psoriasis in adults
Omvoh^TM^ (mirikizumab-mrkz) [12,145]	Humanized mAb (IgG4) (intravenously)	Binds to the p19 subunit of human IL-23 cytokine and inhibits its interaction with the IL-23 receptor	26 October 2023	Eli Lilly and Company, New York, NY, USA	Moderate to severe active ulcerative colitis in adults
Veopoz^TM^ (pozelimab-bbfg) * [146,147]	Human mAb (IgG 4) (intravenously)	Blocks the activity of C5 and prevents diseases mediated by the complement pathway	18 August 2023	Regeneron Pharmaceuticals Inc., Tarrytown, NY, USA	CD55-deficient PLE, also known as Chaple disease in patients aged 1 year and older

This table includes publicly available data from FDA databases such as The Purple Book, The Orange Book, and others. Abbreviations: (*)—Orphan Drug Designation granted by the FDA; IgG—Immunoglobulin; FcRn—Neonatal Fragment Crystalizable Receptor; gMG—Generalized Myasthenia Gravis; AChR—Anti-Acetylcholine Receptor; Anti MuSK—Anti Muscle-Specific Tyrosine Kinase; and IL—interleukin. Importantly, Veopoz^TM^ stands out as the first treatment for Chaple disease, a childhood-onset condition that affects fewer than 100 patients worldwide [148].

## Data Availability

U. S. Food and Drug Administration: Search Orphan Drug Designations and Approvals: https://www.accessdata.fda.gov/scripts/opdlisting/oopd/index.cfm (accessed on 26 June 2024); FDA Purple Book: https://purplebooksearch.fda.gov/results?query=nirsevimab&title=Beyfortus (accessed on 26 June 2024); FDA Orange Book: https://www.fda.gov/drugs/drug-approvals-and-databases/orange-book-data-files (accessed on 26 June 2024); U. S. Food and Drug Administration: Designating an Orphan Product: Drugs and Biological Products: https://www.fda.gov/industry/medical-products-rare-diseases-and-conditions/designating-orphan-product-drugs-and-biological-products (accessed on 26 June 2024).
